# Supplementary material for: Learning curve and surgical outcome of robotic assisted colorectal surgery with ERAS program
Source: Sci Rep. 2022 Nov 29;12:20566. doi: 10.1038/s41598-022-24665-w (PMC9709162; doi:10.1038/s41598-022-24665-w)
Supplement: Supplementary file 2 — Supplementary Information 2. [file 41598_2022_24665_MOESM2_ESM.zip › RAL Raw data-Ñ[▒K/061/HS_ñw╝╨░O▒Kñσ.pdf]

正式摘要(電子簽章：0000002113375000) W55 / W55 ☐ 英文標題(注意:無英文姓名-請補病人基本檔!)	☐【讀取】憑證	

臺中榮民總醫院

出院病歷摘要

轉入號： 轉出號：

[redacted]

**(8)**流水編號	
**(9)**入院日期	110/02/24 (2021) 直腸外科 W55 - 272
**(10)**轉科日期	
**(11)**出院日期	110/03/01 (2021) 住院天數計 5 日

診斷

- 入院：S colon ca
- 出院：S colon tumor s/p robotic AR on 2021/2/25

主訴

- stool OB positive noted for one month

病史

- This patient had no past medical history. The patient had health examination and stool OB was noted in one month. LMD colonoscope was done and showed S colon tumor. The patient came to OPD for further evaluation. Colon scope was repeated and showed 20cm FAV s/p s/p tattoo for localization. CT was done and showed S COLON ca without distal metastasis. This time the patient was admitted for robotic AR.

體檢發現

- ......
  - General appearance :
    - easy looking , conscious clear , without cardiopulmonary distress
    - Vital signs自動帶入date 體溫(BT) 脈搏(P) 呼吸(R) 血壓(BP) 身高(BH) 體重(BW) BMI
      20210224 37C 70/min 18/min 172/90mmHg 167.1cm 59.7Kg 21.4
    - Vital signs: B.P. 136/94 mmHg , P.R. 108 /min , R.R. 18 /min , B.T. 37 ℃
  - HEENT :
    - Eyes: no anemic conjunctiva, no icteric sclera, normal EOM
    - ENT: no otorrhea, no rhinorrhea, no erythematous mucosa, no injected tonsil, no deformity
  - Neck: supple, freely movable, trachea midline,no deformity, no engorged jugular vein , no palpable thyroid or lymph node enlargement
  - Chest:
    - Inspection: Grossly normal, symmetric and fully expansion, no accessory muscle used, no operation scar, no spider angiomata, no gynecomastia
    - Palpation: no palpable mass or nodule, no subcutaneous emphysema, no tenderness
    - Ausculation: clear breathing sound
  - Heart:
    - Percussion: normal heart size
    - Ausculation: regular heart beat, normal S1, S2, no gallop, no murmur
  - Abdomen:
    - Inspection: in
      - flat contour, symetrically, no scar, no stria, no dilated veins, no rashes
    - Ausculation: normoactive bowel sound, no abdominal bruits
    - palpation: soft, no muscle guarding or rigidity, no local tenderness, no rebounding pain, no palpable mass, no palpable pulsation
    - Percussion: no shifting dulllness, no hepatomegaly, no splenomagaly
  -

Peripheral pulsation :	CA	BA	RA	FA	PA	PTA	DPA
Right side :	++	++	++	++	++	++	++/+++
Left side :	++	++	++	++	++	++	++/+++

  - Genito-anus: no tenderness or palpable mass lesion or rectal shelf, normal external genitalia, normal anal tone, brown stool

CRS CONDITION

-
  -
    - Lung

手術日期及方法

- 手術日期及方法20210225 Robot Assisted Colectomy(L)

住院治療經過

- Aftter admission, pre- op survey was done and robotic AR was arranged. Foley was removed on 2/25 and bland one diet was tried. The patient tolerate diet well and had flattus. Under stable condition, the patient was dsicharge with OPD follow up.

合併症

一般檢查

- 累積報告 :

DATE	TIME	ALB	TP	BIL.T	BIL.D	ALKP	AST	ALT	LDH	CHOL	TG	HDL-C	LDL	UA	NH3	AMY
1100224	2040		7.8							344	180	61	244	4.3		
1100225	1307			0.52	0.06		30	17								
1100227	1208			0.27	0.08		17	11								

DATE	TIME	WBC	RBC	HGB	HCT	MCV	PLT	BLST	NEUT	BAND	SEG	LYM	MON	EOS	BAS
1100225	1308	12130		11.5	33.8		266		91.0			7.1	1.6	0.0	0.3
1100227	1209	8570		9.1	27.9		211		78.4			15.1	5.1	0.9	0.5

DATE	TIME	NA	K	CL	CA	BUN	CREAT	Mg	P	CRP	ESR	PCT	LIPASE	LACTATE
1100224	2040									0.032				
1100225	1307	140	3.8			11	0.61							
1100227	1208	141	3.6			6	0.71							11.6

DATE	TIME	SP-GR	PH	PROT	GLUC	KETON	BILI	UROB	OB	NITR	LEU	RBC	WBC	EPCEL
1100227	1229	1.007	7.0	Negative	Negative	Negative	Negative	0.2	Negative	Negative	Negative	0-2	0-2	0-1

特殊檢查

病理報告

其它

出院時情況

- 3.改門診治療

出院指示

-

藥名	劑量	劑量單位	用法	頻次	數量	數量單位	現況	天數	單位
$Biofermin tab	2	TAB	PO	TID	84	TAB	TH	14	天
Acetal tab 500mg	1	TAB	PO	Q6HPRN	28	TAB	TH	7	天
(□)Takepron OD tab 30mg	1	TAB	PO	QDAC	14	TAB	TH	14	天

出院計劃

主治醫師 2062F 蔣鋒帆 / 住院醫師 2332G 林忠安

正式摘要(**電子簽章：000000211375000)**

W55 / W55

doctorRank:B
